# Oncocytic carcinoma of parotid gland: a case report with clinical, immunohistochemical and ultrastructural features

**DOI:** 10.1186/1477-7819-4-54

**Published:** 2006-08-21

**Authors:** Giovanna Giordano, Marzio Gabrielli, Letizia Gnetti, Teore Ferri

**Affiliations:** 1 Department of Pathology and Medicine Laboratory, Section of Pathology, Parma University, Italy; 2 Department of Otolaryngology, Parma University, Italy

## Abstract

**Background:**

Oncocytic carcinoma is an extremely rare neoplasm of the salivary glands. We report a case of oncocytic carcinoma arising in a parotid gland in a 66-year-old female.

**Method:**

An excisional biopsy of the parotid tumor was performed. The specimen was submitted for histology and after fixation in formalin solution and inclusion in paraffin, 3–5 μm sections were stained with hematoxylin and eosin for conventional evaluation and Periodic acid Schiff stain. Immunohistochemical studies were performed using antibodies against mitochondrial antigen, keratin, S-100, alpha-actin, vimentin, alpha-1-antichymotrypsin as well as an ultrastructural analysis was performed.

**Results:**

Frozen sections revealed an infiltrative growth pattern and the diagnosis of a malignant epithelial lesion was made. Permanent sections stained with haematoxylin and eosin revealed a neoplasm that had replaced a wide area of the parotid gland and had invaded subcutaneous adipose tissue. Perineural invasion was evident, but vascular invasion was not found. Neoplastic elements were large, round or polyhedral cells and were arranged in solid sheets, islands and cords. The cytoplasm was abundant, eosinophilic and finely granular. The nuclei were large and located centrally or peripherally. The nucleoli were distinct and large. Periodic acid Schiff stain demonstrated a granular cytoplasm. Immunohistochemistry demonstrated mithochondrial antigen, keratin, and chymotrypsin immunoreactivity in the neoplastic cells. Ultrastructural analysis revealed numerous mitochondria packed into the cytoplasm of the neoplastic cells. Thus, the final diagnosis was that of oncocytic carcinoma of parotid gland.

**Conclusion:**

This neoplasm shows clinical, microscopical, histological and ultrastructural features of oncocytic carcinoma and this must be considered in the differential diagnosis of other proliferations in the parotid gland with abundant granular cytoplasm and metastatic oncocytic carcinomas.

## Background

The occurrence of oncocytic carcinoma of the parotid gland is rare. A new case of oncocytic carcinoma in a parotid gland has been reported recently by Guclu *et al *[1]. According to a review of the literature performed by these authors, only 41 cases have been reported [1]. We report a case of oncocytic carcinoma of the parotid gland with its clinical manifestations and pathological features.

## Case presentation

A 66-year-old female was admitted to our Institution with a history of a painless left preauricular nodule that had gradually increased in size. Computed tomographic (CT) scan revealed a 2 × 2.5 cm solid lesion in the left parotid gland. Cervical and peri-aortic lymph nodes were not enlarged, except for one in the submandibular region.

Total parotidectomy with preservation of the facial nerve was performed. Thus, the parotid gland and covering skin were removed. Lateral jugular lymph nodes dissection was carried out.

The lesion was initially examined in frozen sections. The specimen was submitted for histology and after fixation in formalin solution and inclusion in paraffin, 3–5 μm sections were stained with haematoxylin and eosin for conventional evaluation and a Periodic acid Schiff stain also carried out. A panel of immunostains, including antibodies against mitochondrial antigen, keratin (Citok AE1, Citok AE3), carcinoembryonal antigen (CEA), vimentin, alpha-1-antichymotrypsin, smooth muscle actin and S-100, was applied to representative sections of the tumour using the avidin-biotin complex technique (Table 1). Formalin-fixed small fragments of neoplasm were also examined by electron microscopy, after washing in 0,1 M phosphate buffer, postfixation in osmium tetroxide, dehydratation in ethanol and embedding in epon-araldite.

**Table 1:** Primary antibodies used for immunophenotyping

***Antibody***	***Manufacturer***	***Dilution***	***Method***
Mitochondrial antigen	BioGenex	1:500	ABC
Citok AE_1_/AE_3_	Dako	1:100	ABC
CEA	Dako	1: 25	ABC
Vimentin	Neomarkers	1:500	ABC
Alpha-1-anticymotrypsin	Dako	1: 800	ABC
Smooth muscle actin	Neomarkers	1:500	ABC
S100 protein	BioGenex	1:500	ABC

Ultrathin sections were stained with uranyl acetate and lead citrate and examined with a Philips EM 208 electronic microscope.

## Results

**Figure 1:**
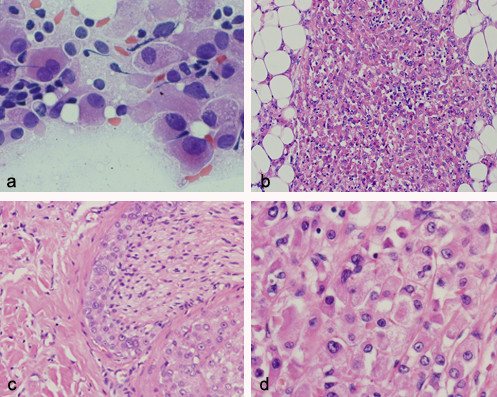
Imprint cytology of lesion showing cohesive clusters of neoplastic cells with abundant and finely granular cytoplasm and moderately pleomorphic nuclei located centrally or peripherally (a: haematoxylin- eosin, × 400). Permanent sections revealed a neoplasm that had invaded subcutaneous adipose tissue (b: haematoxylin- eosin, × 100) and perineural tissue (c: haematoxylin-eosin, × 200). Neoplastic elements with abundant granular eosinophilic cytoplasm, large nuclei and evident nucleoli, are large, round or polyhedral cells arranged in solid sheets, islands and cords (d: haematoxylin-eosin, × 400).

Macroscopically, the tumour was a well-circumscribed, firm, grey-brown, ovoid nodule measuring 2.5 cm in diameter. Imprint cytology of the lesion showed cohesive clusters of neoplastic cells. The cytoplasm was abundant and finely granular. The nuclei were moderately pleomorphic, medium or large and were located centrally or peripherally (Figure 1a). Frozen section revealed an infiltrative growth pattern and the diagnosis of a malignant epithelial lesion was made.

Permanent sections stained with haematoxylin and eosin revealed that the neoplasm that had replaced a wide area of the parotid gland and had invaded subcutaneous adipose tissue (figure 1b). Perineural invasion was evident (figure 1c), but vascular invasion was not found. Neoplastic elements were large, round or polyhedral cells and were arranged in solid sheets, islands and cords. The cytoplasm was abundant, eosinophilic and finely granular. The nuclei were large and located centrally or peripherally. The nucleoli were distinct and large (figure 1d). Periodic acid Schiff stain demonstrated a granular cytoplasm.

**Figure 2:**
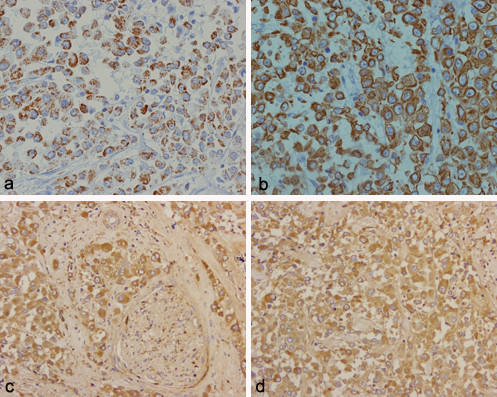
All tumour cells on immunohistochemical study showed finely granular immunoreactivity of the cytoplasm with antimitochondrial antibody (a: ×200), cytoplasmatic and membranous positivity for antikeratin antibody (b: ×200) and cytoplasmatic positivity for alpha-1-antichymotripsina (c and d: × 200; in c yet note neoplastic perineural infiltration).

Immunohistochemically, the tumour strongly reacted with mithochondrial antigen (Figure 2a), keratin (Figure 2b), alpha-1-antichymotrypsin (Figure 2c and 2d), but was negative for smooth muscle actin, vimentin and carcinoembryonal antigen (CEA) and S-100 protein (S-100). All lymph nodes examined were negative for metastases.

On ultrastructural examination, the cytoplasm of neoplastic cells seemed to be packed with numerous mitochondria. These organuli were not clear, since their cristae appeared fragmented because of fixation in formalin solution without adjusted pH.

## Discussion

Oncocytic carcinoma is an extremely rare malignancy in salivary glands, accounting only for 11% of all oncocytic salivary gland neoplasms, 0.5% of all epithelial salivary gland malignancies and 0.18% of all epithelial salivary gland tumours [2]. This neoplasm is characterized by epithelial cells with abundant eosinophilic and granular cytoplasm, filled with numerous mitochondria.

Malignant oncocytoma, malignant oxyphilic adenoma and oncocytic adenocarcinoma have been used synonymously for oncocytic carcinoma. The malignant nature of the neoplasm can be recognized by its morphologic features and infiltrative growth. Morphologic criteria for the diagnosis of a malignant nature are cellular pleomorphism, necrosis and frequent mitoses. Infiltrative growth of the neoplasm is represented by perineural, vascular or lymphatic invasion, destruction of adjacent structures and local lymph node metastasis.

Immunohistochemical study and ultrastructural examination are essential ancillary studies necessary for a correct diagnosis of oncocytic carcinoma to be made; these procedures show the presence of abundant mitochondria in cytoplasm.

In our case, the malignant nature of the neoplasm was evidenced by the presence of perineural invasion and by infiltration of subcutaneous tissue. No regional or distant lymph node metastases clinically or radiologically were observed.

Oncocytic differentiation of neoplastic cells was demonstrated by immunohistochemical positivity for mithochondrial antigen [3], keratin, alpha-1-antichymotrypsin [4]. On ultrastructural analysis numerous mitochondria seemed to fill the cytoplasm. These organuli were not clear because of fixation of tissue for light microscopy, which is similar to the case reported by Mizutary *et al *[5]. Other neoplasms that arise from the salivary gland with a granular cytoplasm are oncocytoma, acinic cell adenocarcinoma and salivary duct carcinoma [6].

Oncocytic carcinoma can be differentiated from benign oncocytoma by the presence of a connective tissue capsule in the latter. Moreover, compared to oncocytoma, oncocytic carcinoma usually shows a greater mitotic activity and more nuclear pleomorphism.

Acinic cell adenocarcinoma can be differentiated from oncocytic carcinoma since its cytoplasmic granules are amphophilic or basophilic. Moreover, the patterns of growth in acinic cell adenocarcinoma can be microcystic or papillary and the neoplastic elements are negative for mithochondrial antigen when examined immunohistochemically. Salivary duct carcinoma, in contrast to oncocytic carcinoma, forms duct-like spaces with papillary and cribriform growth and also shows comedonecrosis [6].

The non-neoplastic proliferation of a salivary gland, which can mimic oncocytic carcinoma is oncocytosis. This lesion is a condition that predominantly affects adults over the age of 60 years, and can be differentiated from malignant oncocytoma by the presence of variably sized foci of oncocytic cells within glandular lobules without altering the normal architecture of the gland [6].

Primary oncocytic carcinoma of the salivary glands should also be differentiated from metastatic oncocytic carcinomas to the salivary glands from a precise clinical history, revealing the previous primary neoplasm and by specific immunohistochemical studies.

Metastatic oncocytic carcinoma of the thyroid (Hürthle cell carcinoma) can be diagnosed because of the immunohistochemical expression of thyroglobulin [7].

The diagnosis of a rare metastatic oncocytic adenocarcinoma of the stomach to the salivary gland is facilitated by the presence of a tubular pattern of growth and by the presence of microvilli on the luminal surfaces of neoplastic cells, which are absent in salivary oncocytic carcinoma of the salivary gland [8].

Metastatic renal cell carcinoma, the granular type, must be considered in the differential diagnosis of primary oncocytic carcinoma in a salivary gland. This variant of renal carcinoma is characteristically composed of cells organized in sheets, cords or as papillary fronds [9]. On immunohistochemical examination, oncocytic carcinoma in a salivary gland is negative for carcinoembryonal antigen (CEA) and S-100 protein [10] in contrast with renal carcinoma [11,12] which is positive to either markers. In our case, the diagnosis of primary oncocytic carcinoma of parotid gland was made by immunohistochemical analysis revealing negativity for S100 and CEA according to other studies reported in the literature [10] and by a negative clinical history for a renal tumour.

The prognosis of oncocytic carcinoma in salivary gland is not well known, because of its rarity. Goode and Corio have reported that tumours smaller than 2 cm in diameter appeared to have a better prognosis than those that were larger [13].

In our case, the neoplasm was 2.5 cm in diameter and was not associated with local or distant metastases. A good prognosis is expected for our patient, because there was not involvement of the lymph nodes.

## Conclusion

Oncocytic oncocytic carcinoma may have similar morphologic features with other neoplasms. However histological, immunohistological and ultrastructural features will aid in differentiating one from the other.

## Competing interests

The author(s) declare that they have no competing interests.

## Authors' contributions

GG: Wrote the manuscript and did the pathological work-up

MG: Did the pathological work-up and prepared photomicrographs

LG: reviewed the literature and helped in preparation of manuscript

TF: operated on the patient and revised the manuscript
